# TNF-α Mediates the Association between Dietary Inflammatory Index and Depressive Symptoms in Breast Cancer

**DOI:** 10.3390/nu15010084

**Published:** 2022-12-24

**Authors:** Yue Chen, Gusonghan Maitiniyazi, Ziyuan Li, Tong Li, Yuan Liu, Rong Zhang, Xiaoyun Cao, Danfeng Gu, Shufang Xia

**Affiliations:** 1 Wuxi School of Medicine, Jiangnan University, Wuxi 214122, China; 2 Department of Psychiatry, Jiangsu Rongjun Hospital, Wuxi 214035, China; 3 Department of Breast Surgery, Affiliated Hospital of Jiangnan University, Wuxi 214125, China; 4 Department of Breast Surgery, Affiliated Obstetrics and Gynecology Hospital of Jiangnan University, Wuxi 214002, China

**Keywords:** dietary inflammatory index, breast cancer, depressive symptoms, nutrients, inflammation

## Abstract

This study examined the association between the energy-adjusted Dietary Inflammatory Index (E-DII)-based dietary inflammatory potential and depressive symptoms (DepS) among patients with breast cancer and explores whether systemic inflammation mediates this association. We assessed dietary intake and DepS in 220 breast cancer patients by three 24 h dietary recalls and the Center for Epidemiological Studies Depression Scale (CES-D), respectively, and determined plasma levels of C-reactive protein (CRP), tumor necrosis factor-α (TNF-α), and interleukin (IL)-1β, IL-4, and IL-6 in 123 blood samples. We found that each one-point increase of E-DII was related to a 53% elevated risk of DepS. Patients with the most pro-inflammatory diets had a 5.13 times higher risk of DepS than those with the most anti-inflammatory diets. Among the E-DII components, vitamin B_2_, zinc, and iron were inversely associated with DepS risk. Furthermore, E-DII scores were positively associated with CRP and TNF-α. Higher levels of TNF-α and IL-6 were associated with higher DepS risk. A significant mediating effect of TNF-α was revealed between E-DII and DepS. Our findings suggest that a pro-inflammatory diet is positively associated with breast cancer-related DepS, which may be mediated by TNF-α.

## 1. Introduction

Breast cancer has become the most prevalent cancer and the fifth largest cause of cancer-induced death among females worldwide, with 2.26 million new cases and 685,000 deaths recorded globally in 2020 [1]. Due to its incurable nature and long-term exposure to the disease, breast cancer imposes enormous physical symptoms and psychological responses on patients [2]. Among them, emotional distress is regarded as the sixth vital sign of cancer care due to its multiple negative effects on the treatment and prognosis of cancer patients [3]. As a kind of emotional distress, depressive symptoms (DepS) are relatively common among breast cancer patients, with 9.4% to 66.1% prevalence [4], and yet are frequently overlooked, leading to diminished quality of life, decreased treatment compliance and poorer physical functioning [5]. It has been suggested that cancer patients with comorbid depression experience worse fatigue, pain, anxiety, and functioning impairment than those with cancer only, and are more prone to have suicide thoughts [6]. Recently, DepS has been established as an independent factor in predicting breast cancer recurrence and survival [7]. However, the reality is that nearly 73% of depressed breast cancer patients are undertreated [6]. The current treatment for depression is unsatisfactory because of its high cost, limited therapeutic efficacy and adverse side effects [8]. Therefore, effective interventions for breast cancer-related DepS are urgently needed.

Although medication and psychological interventions are the first-line therapies for depression, lifestyle intervention (including diet and physical activity) is mutable, safe and low-cost, and may provide the basis of practical interventions to manage depression. Among the lifestyle factors, the diet has been considered as the central determinant of mental health [9]. The relationship between depression and diet has been verified both in prospective and cross-sectional studies. It has been suggested that unhealthy dietary patterns, such as the Western diet, are linked with an increased depression risk, while healthy dietary patterns, such as the Mediterranean diet, can reduce depression risk [10]. Individual nutrients are also related to depression. For example, total fiber, as well as insoluble, vegetable, fruit, and cereal fiber, is correlated with lower DepS in healthy populations [11]. However, due to the lack of professional dietary guidance [12], most breast cancer patients have unreasonable dietary patterns, poor diet quality and insufficient nutrients intake, which is significantly associated with DepS [13].

Despite the fact that the mechanisms revealing the link between diet and depression are not totally explained, several biological processes have been postulated in which diet might act on inflammation, oxidative stress and brain plasticity, leading to depression [14]. Accumulated evidence suggested that inflammation might be involved in the association between diet and depression [15]. A western dietary pattern rich in red meat, refined foods and confectionary is considered as a pro-inflammatory diet and related to an elevated risk of DepS, whereas a healthy dietary pattern characterized by more intake of whole grains, fruits, vegetables, and fish, appears protective in inflammation and DepS [16]. Additionally, dietary micronutrients have also been associated with depression through regulating inflammation. Omega-3 fatty acids are protective against depression in older men by decreasing C-reactive protein (CRP) levels, while the total intake of fat, saturated fatty acids (SFA), and monounsaturated fatty acids (MUFA) exerted an increased effect on both CRP and interleukin (IL)-6 in older women [17]. In breast cancer patients, positive correlations have been observed between depression and inflammatory markers, such as IL-1β, IL-6, and tumor necrosis factor-alpha (TNF-α) [18]. A longitudinal study also revealed that the acid-producing diets were positively associated with DepS in breast cancer survivors [19]. To date, no studies have assessed the inflammatory potential of the diet on systemic inflammation and DepS in breast cancer patients.

It is well-accepted that diet is a complex set of exposures which interact with each other, and whose accumulated impact regulates inflammatory reactions as well as health outcomes. The dietary inflammatory index (DII), a literature-derived and population-based index, was developed to evaluate the inflammatory potential of an individual’s diet [20]. Previous research has illustrated a positive association between DII and depression, among the general population rather than cancer patients [21]. Several studies have attempted to explore the association between DII-assessed diet and depression, with inflammatory markers as mediators, but limited findings have been revealed [22]. Therefore, the aim of this study was to examine the association between energy-adjusted DII (E-DII) and DepS in breast cancer patients, as well as the mediating role of systemic inflammatory markers.

## 2. Materials and Methods

### 2.1. Participants and Study Design

All participants in this cross-sectional study were recruited at the Affiliated Hospital of Jiangnan University and Wuxi Maternal and Child Health Care Hospital from September 2021 to February 2022. Participants were included based on the following inclusion criteria: female breast cancer without metastasis; aged from 20 to 80 years old; normal cognitive function and reading ability; and willingness to participate in this research. The exclusion criteria were: previously diagnosed with other cancers; prior physician-diagnosed psychiatric disorders before or after breast cancer diagnosis; diagnosed with hematological, immune or inflammatory diseases; use of antidepressants and anxiolytic medications; pregnancy or lactation; use of radiotherapy, chemotherapy drugs, antibiotics or immune-modulatory medications in the past 21 days; incomplete questionnaires or clinical data. A total of 243 subjects diagnosed with breast cancer were initially enrolled, of whom 23 individuals were excluded according to the exclusion criteria (i.e., 9 subjects were previously diagnosed with other cancers, 2 subjects were using antidepressants, 2 subjects were using antibiotics, and 10 subjects lacked complete dietary information), and finally a total of 220 participants were included in this study.

This study was approved by the Medical Ethics Committee of Jiangnan University (JNU20210918IRB11) and implemented in compliance with the Declaration of Helsinki. All participants provided written informed consent.

### 2.2. Sample Size

The following formula was used to calculate the sample size: *n* = Z^2^_α/2_ (1 − *p*) *p*/δ^2^. Where α = 0.05, Z_α/2_ = 1.96. The prevalence of DepS in Chinese breast cancer patients was 36.4% [23], and the permissible error δ = 0.18*p* = 0.0655. The calculated minimum sample size was 207.

### 2.3. Data Collection and Measurement

Face-to-face interviews were performed with all participants by trained researchers to obtain data on sociodemographic information, physical activity, smoking and drinking status, DepS and anxiety symptoms, and dietary intakes. Clinical data on comorbid chronic diseases, cancer stage and surgery type were collected from medical records.

#### 2.3.1. Assessment of Physical Activity Level

Physical activity level was assessed by the International Physical Activity Questionnaire Short Form (IPAQ-SF) to calculate the metabolic equivalent (MET) [24] and then classified into three levels: low (<600 MET/min/week), moderate (600 to 1500 MET/min/week), and high (>1500 MET/min/week).

#### 2.3.2. Assessment of DepS and Anxiety Symptoms

DepS were evaluated using the Center for Epidemiologic Studies Depression Scale (CES-D) [25], a 20-item self-reported questionnaire, and the criterion validity has been confirmed among breast cancer patients [26], in which a CES-D score ≥16 was designated as depressed patients and a CES-D score <16 was defined as non-depressed patients.

The Chinese version of the Self-Rating Anxiety Scale (SAS), with adequate reliability and validity, was used to evaluate anxiety symptoms [27]. A higher SAS score indicates more severe anxiety symptoms.

#### 2.3.3. Assessment of Dietary Intake and Calculation of E-DII

Dietary intake was estimated through three 24 h dietary recalls, in which participants needed to describe the types and amounts of all the foods and dietary supplements that they had consumed in the past 24 h. The trained researchers used food models and photographs to help participants recall their dietary intake, and participants’ family members or caregivers were recommended to be present to help with recall. The Nutrition Calculator v2.7.8.8 (Institute of Nutrition and Food Safety, Chinese Center for Disease Control and Prevention, Beijing, China) was used to calculate the average nutrients intake from the dietary data. In this study, the following 26 food parameters were output for subsequent DII/E-DII score calculations: energy, total fat, protein, carbohydrates, dietary fiber, cholesterol, vitamins A, B_1_, B_2_, B_6_, B_12_, C, D, and E, folic acid, niacin, β-carotene, magnesium, iron, zinc, selenium, SFA, MUFA, polyunsaturated fatty acids (PUFA), omega-3 and omega-6 fatty acids.

The DII algorithm is the weighted sum (weights are literature-derived inflammatory effect scores) of the standardized values for each participant’s intake of specific food parameters using the common global daily mean intake and standard deviation (SD) deduced from the representative global database. The development and validation of the DII have been explained in detail elsewhere [20,28]. To control for the effect of total energy intake, the E-DII was calculated based on the published literature [29]. First, all dietary intake was converted to values per 1000 kcal. Second, this energy-adjusted dietary intake was standardized as a z-score by subtracting the energy-adjusted global standardized mean and then dividing by the energy-adjusted global SD (Table S1). Third, the z-score of each food parameter was converted to a percentile score, which was then centered on 0 by doubling the values and subtracting 1. Fourth, each centered percentile value was multiplied by its respective parameter-specific inflammatory effect score to obtain the E-DII score of each food parameter. Finally, all food parameter-specific E-DII scores were summed to create the total E-DII score of each participant. Higher E-DII scores of the diet suggest higher pro-inflammatory potential, whereas lower scores suggest higher anti-inflammatory potential.

### 2.4. Assessment of Inflammatory Markers

A total of 123 blood samples were collected after overnight fasting before chemotherapy. Plasma was extracted by centrifugation at 3500 rpm for 10 min and then stored at −80 °C. According to the manufacturer’s instructions, plasma inflammatory biomarkers (TNF-α, CRP, IL-6, IL-1β, and IL-4) were measured by the ELISA method.

### 2.5. Statistical Analysis

Statistical analyses were performed on SPSS 26.0 (IBM SPSS, Inc., Chicago, IL, USA). Continuous data were presented as median (25th, 75th percentile), and categorical variables were presented as frequencies (n) with percentages (%). The Shapiro–Wilk test was used to verify the normality of variables in each group, and non-parametric statistical tests were applied since the data were not normally distributed. The Mann–Whitney U test and Kruskal–Wallis test were performed in continuous variables to explore the significance between groups. The Chi-square test and Fisher’s exact test were used for categorical variables. For those on-study characteristics that differed between depressed and non-depressed, we used univariate logistic regression analysis to further analyze the possible influencing factors of DepS (Table S2), and then the characteristics with *p* < 0.05 in the multivariate analysis were finally considered as covariates in subsequent analyses, including SAS scores and drinking status (Table S3). In addition, as a common confounder, age was also considered as a covariate in the subsequent analyses. Multiple logistic regression analysis was used to investigate the effects of E-DII and its component nutrients on DepS. Linear regression and logistic regression were used to analyze the association of E-DII and its components, or DepS with inflammatory markers, respectively. A value of *p* < 0.05 was considered statistically significant.

The R Mediation package 4.5.0 [30] was adopted to examine the potential mediating effect of TNF-α on the association between E-DII (exposure) and DepS risk (outcome). For this model, the average causal mediation effect (ACME), average direct effect (ADE) and average total effect (ATE) were estimated. The ACME is the indirect effect of exposure on the outcome through a mediator. The ADE is the direct effect of exposure on the outcome, excluding the selected mediator. The ATE equals the sum of the ACME and ADE. The 95% confidence interval (CI) was estimated by non-parametric bootstrapping with 5000 replications. All analyses were controlled for age, SAS scores, and drinking status.

## 3. Results

### 3.1. Participants’ Characteristics

Out of the 220 participants, 60 (27.3%) patients with CES-D scores ≥16 were allocated to the depressed group, and the remaining 160 (72.7%) participants were categorized into the non-depressed group. The characteristics of the participants stratified by CES-D scores were illustrated in Table 1. E-DII scores ranged from –4.72 (most anti-inflammatory score) to +3.72 (most pro-inflammatory score). The depressed group demonstrated significantly increased E-DII scores compared with the non-depressed group (*p* < 0.001). In addition, statistical differences were also found in SAS scores (*p* < 0.001), education level (*p* = 0.036), residence (*p* = 0.040), family monthly income (*p* = 0.031), physical activity level (*p* = 0.012), and drinking status (*p* = 0.017) between the two groups. The above significantly different variables were then included in univariate logistic regression analyses, showing that E-DII scores (OR = 1.50; 95% CI: 1.24, 1.83; *p* < 0.001), SAS scores (OR = 1.33; 95% CI: 1.23, 1.44; *p* < 0.001), education level (OR = 0.39, 95% CI: 0.17~0.86 for middle school compared to primary school or lower; OR = 0.28, 95% CI: 0.11~0.72 for junior college or higher compared to primary school or lower), residence (OR = 0.34, 95% CI: 0.15~0.80 for towns compared to rural areas), family monthly income (OR = 0.30; 95% CI: 0.12~0.78 for >5000 compared to <2000), physical activity level (OR = 0.42; 95% CI: 0.23~0.79 for moderate level compared to low level), and drinking status (OR = 0.14; 95% CI: 0.03~0.74 for never drinking compared to former or current drinking) might all be related to DepS (Table S2). However, in the further multivariate logistic analysis, only E-DII scores (OR = 1.52; 95% CI: 1.16, 1.99; *p* = 0.003), SAS scores (OR = 1.36; 95% CI: 1.23, 1.50; *p* < 0.001) and drinking status (OR = 0.09; 95% CI: 0.01, 0.71; *p* = 0.022) were independent influencing factors of DepS (Table S3).

### 3.2. Nutrients Intake

The nutrients intakes in different E-DII tertiles were presented in Table S4. Carbohydrates and SFA intakes were the lowest in the lowest E-DII tertile, whereas the consumption of dietary fiber, vitamin A, B_2_, B_6_, C, D, and E, folic acid, niacin, β-carotene, magnesium, iron, zinc, selenium, PUFA, omega-3 and omega-6 fatty acids were the lowest in the highest E-DII tertile. The nutrients that significantly differed among the E-DII tertiles above were further analyzed between the depressed and non-depressed patients, and the results were shown in Table 2. The depressed patients had significantly higher intakes of carbohydrates, and lower intakes of vitamin A, B_2_, and B_6_, folic acid, β-carotene, iron, and zinc in relation to non-depressed patients (*p* < 0.05).

### 3.3. Plasma Inflammatory Markers

As demonstrated in Table 3, the CRP, TNF-α, IL-6, and IL-1β levels in the depressed patients were remarkably higher than those of the non-depressed patients (*p* < 0.05), while no significance was found in IL-4 levels between the two groups (*p* = 0.655).

### 3.4. Association between E-DII and DepS

The associations between the E-DII or its components and DepS were shown in Table 4. When E-DII was modelled as a continuous variable, each one-point increase of E-DII was associated with a 53% elevated risk of DepS (OR = 1.53; 95% CI: 1.19, 1.97; *p* = 0.001), and a similar positive correlation was also observed when E-DII was fitted as tertiles, with patients in the most pro-inflammatory tertile being 5.13 times more likely to be depressed than those in the most anti-inflammatory tertile (OR = 5.13; 95% CI: 1.76, 14.96; *p* = 0.002). Significant associations between E-DII components and DepS were only observed in vitamin B_2_ (OR = 0.39; 95% CI: 0.17, 0.91; *p* = 0.029), iron (OR = 0.92; 95% CI: 0.86, 0.98; *p* = 0.005), and zinc (OR = 0.82; 95% CI: 0.72, 0.94; *p* = 0.003).

### 3.5. Associations between E-DII and Inflammatory Markers

Multiple linear regression analysis revealed that E-DII scores were positively associated with CRP (β = 0.29; 95% CI: 0.01, 0.05; *p* < 0.05) and TNF-α (β = 0.22; 95% CI: 0.04, 0.41; *p* < 0.05) (Table S5), while vitamin B_6_, one of the E-DII components, was negatively related with CRP (β = −0.22; 95% CI: −0.46, −0.06; *p* < 0.05).

### 3.6. Associations between Inflammatory Markers and DepS

The results indicated that TNF-α (OR = 1.72; 95% CI: 1.18, 2.50; *p* = 0.005) and IL-6 (OR = 4.80; 95% CI: 2.02, 11.37; *p* < 0.001) were positively correlated with the DepS risk in the breast cancer patients, while no significant associations were found between CRP, IL-1β and DepS risk (*p* > 0.05, Table 5).

### 3.7. Association between E-DII and DepS with TNF-α as a Mediator

**Figure 1:**
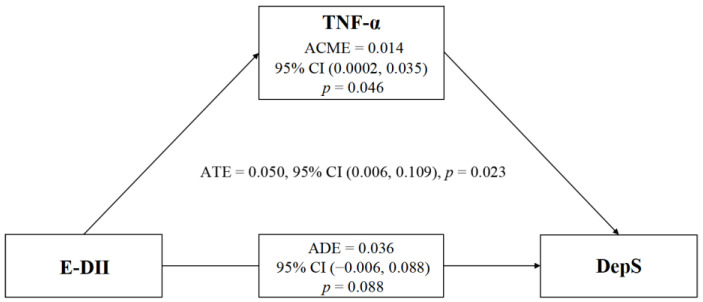
Mediating effect of TNF-α between E-DII and DepS risk after adjusting for age, SAS scores, and drinking status. The 95% confidence interval (CI) in brackets was computed from 5000 nonparametric bootstrap replicates. E-DII, energy-adjusted Dietary Inflammatory Index; TNF-α, tumor necrosis factor α; DepS, depressive symptoms; ACME, average causal mediation effect; ADE, average direct effect; ATE, average total effect.

We performed a mediation analysis to understand whether diet-induced differences in the inflammatory marker TNF-α caused DepS (Figure 1). The results showed that the association between E-DII and DepS was significantly mediated by TNF-α (ACME = 0.014; 95% CI: 0.0002, 0.035; *p* = 0.046).

## 4. Discussion

This cross-sectional study determined the associations between E-DII and its components and DepS in the breast cancer patients, and the mediating effects of inflammatory markers in these associations. We found that the patients with higher E-DII scores had increased risk of DepS, while parts of the E-DII components, including vitamin B_2_, iron and zinc, reduced the risk of DepS. Mediation analysis revealed that TNF-α mediated the association between E-DII and DepS.

DepS is a common mental disorder among breast cancer patients which can negatively affect prognosis, treatment adherence, quality of life, and survival [7]. Recent research has focused on the effect of nutrition in the management of depression [31]. It is well-known that the production of neurotransmitters needs adequate amounts of nutrients, such as minerals (iron, zinc and magnesium), B vitamins (B_6_, B_12_ and folic acid) and amino acids (tyrosine and tryptophan). Most of those mentioned above can be found in whole grains, eggs, beans, yogurt, vegetables and fruits [32]. In this study, we found that, compared with non-depressed breast cancer patients, depressed patients consumed more carbohydrates and less vitamins (vitamin A, B_2_, B_6_ and folic acid), minerals (iron and zinc), and β-carotene, among which vitamin B_2_, iron, and zinc were negatively associated with DepS. It has been suggested that depressed patients often suffer from nutrient deficiencies, including fatty acids, B vitamins, minerals, and amino acids [33], which might be due to the fact that most depressed patients have unhealthy dietary habits, such as increased sweets and high-fat food intake and decreased vegetables, fruits and fish intake [34]. Therefore, adherence to a healthy diet might be effective in decreasing the occurrence of DepS in breast cancer patients.

Diet may influence depression partially by acting on inflammatory pathways [35]. An alleviation in inflammation was related to improved DepS after dietary intervention [36]. Accumulating evidence has indicated that food and nutrients have pro-/anti-inflammatory characteristics which may affect depression, and it was observed that the inflammatory potential of diet was related to an elevated risk of DepS [37]. Results from the Nurses’ Health Study first revealed an association between an inflammatory dietary pattern and a higher depression risk [38]. Nowadays, the DII and E-DII [20] are both essential tools for standardizing the overall inflammatory potential of an individual’s diet, which have been validated among diverse populations and widely used to verify the association between dietary inflammation and depression [22]. Our findings in breast cancer patients further verified the relationship between a pro-inflammatory diet and DepS. Notably, the risk of DepS in the most pro-inflammatory group was 5.13 times that of the most anti-inflammatory group in this study, which was much higher than those previously reported in the general population [21], probably due to the increased susceptibility to inflammation in breast cancer patients experiencing a high-stress environment after disease diagnosis [39]. Given the significant effect of pro-inflammatory diets on DepS, more attention should be paid to forming professional dietary guidelines for breast cancer patients to follow to prevent the occurrence of DepS.

It is well-known that inflammatory cytokines can cross the blood–brain barrier and interact with pathophysiological mechanisms relevant to depression, including the neurotransmitter metabolism, neuroendocrine function and neuroplasticity [40]. Numerous studies have revealed that diet can regulate inflammatory cytokines, since various specific nutrients appear to counteract inflammation [41]. In our research, we selected inflammatory markers previously reported to be associated with depression and found that these plasma inflammatory markers were statistically correlated with E-DII and its components, as well as the occurrence of DepS in breast cancer patients. Specifically, we found that elevated plasma IL-6 and TNF-α levels were associated with higher DepS risk, which was consistent with the previous research in breast cancer patients receiving adjuvant therapy [42]. Additionally, positive associations between IL-1β, TNF-α and DepS were also found in postoperative breast cancer patients untreated with adjuvant chemotherapy [18]. A recent meta-analysis summarized 54 studies and suggested that peripheral blood inflammatory markers (CRP, TNF and IL-6) were significantly related to cancer-related DepS and might be helpful for management of DepS in the cancer patients [43]. It has been extensively reported that DII/E-DII was positively correlated with inflammatory markers CRP and TNF-α [44]. Similar results were observed in our study, providing additional evidence for the regulating role of diet on inflammation. In addition, vitamin B_6_, one of the E-DII components, exhibited a negative correlation with CRP, consistent with the findings from patients with rheumatoid arthritis and elderly men [45,46]. Accordingly, we speculated that a diet with anti-inflammatory potential might be beneficial in alleviating inflammation-related DepS in breast cancer patients.

Mediation analysis revealed that the association between the inflammatory potential of the diet measured by E-DII scores and DepS was mediated by TNF-α. In fact, previous studies have attempted to study the role of circulating inflammatory cytokines (mainly CRP and IL-6) in the association between DII/E-DII and DepS [22]. Only one study demonstrated a statistically significant mediating role of CRP between DII and depression in 10,022 adults aged 20 years and older, but it was not considered to be substantial or clinically significant because of the minimal mediating effect [47]. Our study firstly revealed a significant mediating effect of TNF-α between E-DII and DepS, extending new evidence that inflammation mediates the relationship between diet and depression. TNF-α is a multifunctional pro-inflammatory cytokine that plays a key role in the modulation of immunity and induction of inflammation. Recent meta-analyses have revealed high concentrations of IL-6, TNF-α and CRP in depressed patients as compared with healthy controls [43], but only TNF-α demonstrated a genetic association with major depressive disorders (MDD) among pro-inflammatory cytokines [48]. Moreover, intracranial administration led to DepS in rodents [49]. Accumulated clinical evidence indicated that an increase of the plasma TNF-α was related to the development of DepS, and effective interventions to reduce the effect of TNF-α, such as TNF-α inhibitors, could reverse DepS [50]. Anti-TNF-α treatment for Crohn’s disease also significantly improved the patient’s DepS, which further proved that TNF-α might lead to depression [51]. While the molecular mechanism of TNF-α in regulating depression is unclear, researchers speculated that TNF-α might stimulate the hypothalamic-pituitary-adrenal (HPA) axis, interact with neurotransmitters (5-hydroxytryptamine, dopamine, norepinephrine and glutamate), and modulate neuroplasticity and neuroendocrine function, leading to the development of DepS [52]. Regulation of TNF-α to normal levels might be considered as an alternative therapy or prophylactic strategy for depression. A number of studies have illustrated the potential regulatory role of diet on inflammatory markers, especially TNF-α. A diet targeting a lower omega-6/omega-3 ratio by replacing meat with fish and other vegetable oils with olive oil and rapeseed oil for three days a week caused a remarkable decline in TNF-α [53]. Another randomized clinical trial found a remarkable 34.2% reduction in TNF-α following energy-restricted anti-inflammatory diet therapy, which was more than the decrease in hs-CRP and IL-6 [54]. Furthermore, in postmenopausal women with central obesity, both the Central European diet and the Mediterranean diet intervention for 16 weeks resulted in reduced TNF transcription and lower blood TNF-α contents [55]. The use of dietary interventions, such as the adoption of a more anti-inflammatory diet, might improve depression via inflammation modulation. Several intervention trials suggested that Mediterranean diets could prevent and improve depression [56,57]. Although no studies have measured changes in inflammation following dietary interventions in depression, randomized controlled trials of individual nutritional supplements (nutraceuticals) have provided valuable insights into how dietary nutrition affects depression through inflammatory pathways. Rapaport et al. [58] found that MDD patients with elevated baseline inflammatory markers were significantly more responsive to omega-3 fatty acid treatment (i.e., eicosapentaenoic acid). The antidepressant effect of omega-3 fatty acids by inhibiting inflammation was also confirmed in another randomized controlled trial [59]. This study examined the effect of omega-3 fatty acid supplementation on DepS in patients with hepatitis C being treated with interferon alpha (IFN-α), which commonly induced DepS due to its pro-inflammatory effects. Surprisingly, omega-3 fatty acids reduced the DepS risk following IFN-α treatment. Furthermore, other nutrients, such as folate, have also been found to reduce depression in patients with high levels of inflammation [60], further suggesting these adjunctive treatments may provide symptomatic benefits through anti-inflammation. Overall, our results suggested that TNF-α might mediate the association between diet and DepS, which has an important implication for the prevention and management of DepS.

To our knowledge, the present study is the first to examine the relationship between dietary inflammatory potential and the risk of breast cancer related-DepS and provides significant evidence of the inflammatory marker TNF-α as a mediator. Moreover, we additionally analyzed the effect of nutrient components in E-DII on inflammation and DepS, which is meaningful for further understanding the relationship between diet and mental health. Despite its strengths, this study has certain limitations. First, dietary intake and depression scores were collected by self-report from three 24 h dietary recalls and the CES-D scale, respectively, which might lead to recall bias. Second, patients diagnosed with psychiatric disorders before or after cancer diagnosis were excluded from the study, indicating that we only focused on the mild symptomatology of depression. Third, because it is challenging to completely assess all food intakes in diverse populations [61], the use of more than 20 items from the list of required food parameters to calculate the DII score is allowed. In our study, the E-DII was calculated based on 26 of the 45 food parameters in the original version, since several food parameters are rarely consumed by Chinese breast cancer patients, which might affect the accuracy of E-DII. Finally, given the cross-sectional nature of the study design, the temporal order and causal relationships between E-DII, inflammation and DepS cannot be concluded. Given that mediation analysis assumes a temporal relationship between variables that unfolds over time, the use of cross-sectional data may lead to biased estimates of mediating effects. In contrast, longitudinal data allow for more precise inferences about temporal and potentially causal relationships between variables and within the model, and thus future prospective studies with follow-up are needed to validate our findings and to better understand the temporal sequence and causal relationships of these processes.

## 5. Conclusions

Our findings suggested that E-DII scores were positively associated with DepS risk among breast cancer patients, which might be mediated by TNF-α. Therefore, patients who may be at risk for cancer-related DepS should be encouraged to consume more anti-inflammatory foods and fewer pro-inflammatory foods. Further studies, especially prospective studies, are urgently needed to validate the association between diet and DepS in the perspective of inflammation in cancer patients, which might provide useful dietary interventions for the prevention and treatment of DepS.

## Figures and Tables

**Table 1:** Characteristics of the depressed and non-depressed breast cancer patients.

Variables	Depressed (*n* = 60)	Non-Depressed (*n* = 160)	*p*-Value
Age (years) ^1^	52.5 (45.0, 64.8)	53.0 (46.0, 59.0)	0.648
BMI (kg/m^2^) ^1^	23.2 (21.0, 25.7)	23.4 (21.5, 25.3)	0.654
E-DII score ^1^	0.65 (−0.30, 1.97)	−0.61 (−1.41, 0.82)	<0.001
SAS score ^1^	41.9 (37.8, 45.0)	32.5 (30.0, 36.3)	<0.001
Menopausal status, *n* (%) ^2^			
Pre-menopausal	25 (41.7)	64 (40.0)	0.823
Post-menopausal	35 (58.3)	96 (60.0)	
Marital status, *n* (%) ^2^			
Married	53 (88.3)	144 (90.0)	0.719
Widowed/divorced/single	7 (11.7)	16 (10.0)	
Education level, *n* (%) ^2^			
Primary school or lower	17 (28.3)	20 (12.5)	0.036
Middle school	23 (38.3)	70 (43.8)	
High school/ secondary school	10 (16.7)	28 (17.5)	
Junior college or higher	10 (16.7)	42 (26.3)	
Employment, *n* (%) ^2^			
Unemployed	21 (35.0)	48 (30.0)	0.599
Employed	19 (31.7)	47 (29.4)	
Retired	20 (33.3)	65 (40.6)	
Residence, *n* (%) ^2^			
Rural areas	24 (40.0)	41 (25.6)	0.040
Towns	10 (16.7)	50 (31.3)	
Urban areas	26 (43.3)	69 (43.1)	
Family monthly income (RMB), *n* (%) ^2^			
<2000	11 (18.3)	14 (8.8)	0.031
2000–5000	32 (53.3)	74 (46.3)	
>5000	17 (28.3)	72 (45.0)	
Cancer stage, *n* (%) ^2^			
I	13 (21.7)	37 (23.1)	0.511
II	29 (48.3)	87 (54.4)	
III	18 (30.0)	36 (22.5)	
Surgery type, *n* (%) ^2^			
Lumpectomy	17 (28.3)	50 (31.3)	0.675
Mastectomy	43 (71.7)	110 (68.8)	
Presence of comorbidities, *n* (%) ^2^			
No	39 (65.0)	119 (74.4)	0.169
Yes	21 (35.0)	41 (25.6)	
Physical activity level, *n* (%) ^3^			
Low	28 (46.7)	42 (26.3)	0.012
Moderate	31 (51.7)	110 (68.8)	
High	1 (1.7)	8 (5.0)	
Smoking status, *n* (%) ^3^			
Never	58 (96.7)	160 (100)	0.073
Former/current	2 (3.3)	0 (0.0)	
Drinking status, *n* (%) ^3^			
Never	55 (91.7)	158 (98.8)	0.017
Former/current	5 (8.3)	2 (1.3)	

Data are shown as *n* (%) or median (25th, 75th percentile). ^1^ Mann–Whitney U test; ^2^ Chi-square test; ^3^ Fisher’s exact test. BMI, body mass index; E-DII, energy-adjusted Dietary Inflammatory Index; SAS, Self-Rating Anxiety Scale.

**Table 2:** Dietary nutrient intakes of the depressed and non-depressed breast cancer patients.

Variables	Depressed (*n* = 60)	Non-Depressed (*n* = 160)	*p*-Value
Carbohydrate (g/d)	186.5 (152.9, 247.3)	169.5 (133.9, 219.3)	0.018
Dietary fiber (g/d)	11.8 (8.3, 17.8)	12.6 (8.8, 17.1)	0.642
Vitamin A (μgRAE/d)	379.0 (274.3, 642.0)	542.0 (363.0, 729.0)	0.007
Vitamin B_2_ (mg/d)	1.0 (0.7, 1.4)	1.1 (0.8, 1.6)	0.019
Vitamin B_6_ (mg/d)	0.1 (0.1, 0.2)	0.2 (0.1, 0.3)	0.042
Vitamin C (mg/d)	136.1 (73.8, 237.3)	166.6 (95.7, 229.6)	0.152
Vitamin D (μg/d)	0.9 (0.1, 3.0)	1.3 (0.0, 5.2)	0.272
Vitamin E (mg/d)	24.9 (16.1, 31.3)	25.8 (20.4, 33.4)	0.286
Folic acid (μg/d)	124.1 (62.8, 174.7)	144.2 (89.2, 196.2)	0.033
Niacin (mg/d)	12.2 (9.8, 18.3)	14.8 (11.4, 19.6)	0.055
β-Carotene (mg/d)	1.2 (0.4, 2.0)	1.7 (1.0, 2.9)	0.005
Magnesium (mg/d)	286.5 (216.5, 354.0)	313.0 (224.0, 408.0)	0.059
Iron (mg/d)	16.8 (12.2, 20.6)	19.1 (14.6, 23.4)	0.029
Zinc (mg/d)	8.5 (6.2, 11.6)	9.9 (7.8, 13.5)	0.012
Selenium (μg/d)	42.2 (26.5, 78.9)	58.5 (33.5, 90.9)	0.053
SFA (g/d)	15.3 (11.1, 19.3)	14.6 (11.1, 18.8)	0.359
PUFA (g/d)	14.2 (9.8, 18.7)	14.6 (10.0, 18.0)	0.454
Omega-3 fatty acids (g/d)	0.2 (0.1, 0.2)	0.2 (0.1, 0.3)	0.707
Omega-6 fatty acids (g/d)	1.3 (0.8, 1.6)	1.2 (0.8, 1.6)	0.454

Data are shown as median (25th, 75th percentile). The nutrients analyzed here were statistically different among E-DII tertiles. E-DII, energy-adjusted Dietary Inflammatory Index; SFA, saturated fatty acids; PUFA, polyunsaturated fatty acids.

**Table 3:** Plasma inflammatory markers in depressed and non-depressed breast cancer patients.

Variables	Depressed (*n* = 34)	Non-Depressed (*n* = 89)	*p*-Value
CRP (mg/L)	1.89 (1.78, 2.04)	1.82 (1.69, 1.92)	0.006
TNF-α (pg/mL)	12.89 (12.30, 14.09)	12.25 (10.60, 13.08)	0.001
IL-6 (pg/mL)	7.23 (6.62, 7.63)	6.20 (5.42, 6.81)	<0.001
IL-1β (pg/mL)	11.25 (10.62, 11.84)	10.57 (9.52, 11.59)	0.010
IL-4 (pg/mL)	6.67 (6.15, 7.05)	6.69 (6.10, 7.01)	0.655

Data are shown as median (25th, 75th percentile). CRP, C-reactive protein; TNF-α, tumor necrosis factor α; IL-6, interleukin 6; IL-1β, interleukin 1β; IL-4, interleukin 4.

**Table 4:** Association between the E-DII and its components with depressive symptoms (*n* = 220).

Variables	OR	95% CI	*p*-Value
E-DII (Continuous)	1.53	1.19, 1.97	0.001
E-DII (Categorical)			
Lowest tertile (−4.72~−0.92)	1.00	Reference	0.002 *
Middle tertile (−0.91~0.63)	1.75	0.59, 5.21	
Highest tertile (0.64~3.72)	5.13	1.76, 14.96	
Nutrients			
Carbohydrate	1.00	0.99, 1.01	0.863
Vitamin A	1.00	0.99, 1.00	0.341
Vitamin B_2_	0.39	0.17, 0.91	0.029
Vitamin B_6_	0.07	0.01, 1.03	0.053
Folic acid	1.00	0.99, 1.00	0.224
β-Carotene	0.84	0.66, 1.08	0.170
Iron	0.92	0.86, 0.98	0.005
Zinc	0.82	0.72, 0.94	0.003

The E-DII components analyzed here were statistically different between depressed and non-depressed groups and among E-DII tertiles. Multiple logistic regression was used after adjusting for age, SAS scores and drinking status. E-DII, energy-adjusted Dietary Inflammatory Index. * *p*-value for trend derived using the median approach.

**Table 5:** Association between plasma inflammatory markers and depressive symptoms (*n* = 123).

Variables	OR	95% CI	*p*-Value
CRP	11.40	0.74, 176.52	0.082
TNF-α	1.72	1.18, 2.50	0.005
IL-6	4.80	2.02, 11.37	<0.001
IL-1β	1.46	0.96, 2.22	0.081

Multiple logistic regression analysis was used after adjusting for age, SAS scores and drinking status. CRP, C-reactive protein; TNF-α, tumor necrosis factor α; IL-6, interleukin 6; IL-1β, interleukin 1β.

## Data Availability

The data presented in this study are available from the corresponding author upon request.

## Supplementary Materials

The following supporting information can be downloaded at: https://www.mdpi.com/article/10.3390/nu15010084/s1, Table S1: Food parameters of energy-adjusted dietary inflammatory index (E-DII) used in this study; Table S2: Univariate logistic regression analysis of factors influencing depressive symptoms in breast cancer patients; Table S3: Multivariate logistic regression analysis of factors influencing depressive symptoms in breast cancer patients; Table S4: Dietary nutrient intakes of breast cancer patients in different E-DII tertiles; Table S5: Association between E-DII and its components with plasma inflammatory markers.

## References

[1] Sung H., Ferlay J., Siegel R.L., Laversanne M., Soerjomataram I., Jemal A., Bray F. (2021). Global cancer statistics 2020: GLOBOCAN estimates of incidence and mortality worldwide for 36 cancers in 185 countries. CA Cancer J. Clin..

[2] Würtzen H., Dalton S.O., Elsass P., Sumbundu A.D., Steding-Jensen M., Karlsen R.V., Andersen K.K., Flyger H.L., Pedersen A.E., Johansen C. (2013). Mindfulness significantly reduces self-reported levels of anxiety and depression: Results of a randomised controlled trial among 336 Danish women treated for stage I–III breast cancer. Eur. J. Cancer.

[3] Linden W., Vodermaier A., Mackenzie R., Greig D. (2012). Anxiety and depression after cancer diagnosis: Prevalence rates by cancer type, gender, and age. J. Affect. Disord..

[4] Maass S.W., Roorda C., Berendsen A.J., Verhaak P.F., de Bock G.H. (2015). The prevalence of long-term symptoms of depression and anxiety after breast cancer treatment: A systematic review. Maturitas.

[5] Lan B., Jiang S., Li T., Sun X., Ma F. (2020). Depression, anxiety, and their associated factors among Chinese early breast cancer in women under 35 years of age: A cross sectional study. Curr. Probl. Cancer.

[6] Walker J., Hansen C.H., Martin P., Symeonides S., Ramessur R., Murray G., Sharpe M. (2014). Prevalence, associations, and adequacy of treatment of major depression in patients with cancer: A cross-sectional analysis of routinely collected clinical data. Lancet Psychiatry.

[7] Wang X., Wang N., Zhong L., Wang S., Zheng Y., Yang B., Zhang J., Lin Y., Wang Z. (2020). Prognostic value of depression and anxiety on breast cncer recurrence and mortality: A systematic review and meta-analysis of 282,203 patients. Mol. Psychiatry.

[8] Mauskopf J.A., Simon G.E., Kalsekar A., Nimsch C., Dunayevich E., Cameron A. (2009). Nonresponse, partial response, and failure to achieve remission: Humanistic and cost burden in major depressive disorder. Depress. Anxiety.

[9] Sarris J., Logan A.C., Akbaraly T.N., Paul Amminger G., Balanzá-Martínez V., Freeman M.P., Hibbeln J., Matsuoka Y., Mischoulon D., Mizoue T. (2015). International Society for Nutritional Psychiatry Research consensus position statement: Nutritional medicine in modern psychiatry. World Psychiatry.

[10] Quirk S.E., Williams L.J., O’Neil A., Pasco J.A., Jacka F.N., Housden S., Berk M., Brennan S.L. (2013). The association between diet quality, dietary patterns and depression in adults: A systematic review. BMC Psychiatry.

[11] Swann O.G., Kilpatrick M., Breslin M., Oddy W.H. (2020). Dietary fiber and its associations with depression and inflammation. Nutr. Rev..

[12] Vuksanovic D., Sanmugarajah J., Lunn D., Sawhney R., Eu K., Liang R. (2021). Unmet needs in breast cancer survivors are common, and multidisciplinary care is underutilised: The Survivorship Needs Assessment Project. Breast Cancer.

[13] Yeter K., Rock C.L., Pakiz B., Bardwell W.A., Nichols J.F., Wilfley D.E. (2006). Depressive symptoms, eating psychopathology, and physical activity in obese breast cancer survivors. Psychooncology.

[14] Sarris J., O’Neil A., Coulson C.E., Schweitzer I., Berk M. (2014). Lifestyle medicine for depression. BMC Psychiatry.

[15] Molendijk M., Molero P., Ortuño Sánchez-Pedreño F., Van der Does W., Angel Martínez-González M. (2018). Diet quality and depression risk: A systematic review and dose-response meta-analysis of prospective studies. J. Affect. Disord..

[16] Oddy W.H., Allen K.L., Trapp G.S.A., Ambrosini G.L., Black L.J., Huang R.C., Rzehak P., Runions K.C., Pan F., Beilin L.J. (2018). Dietary patterns, body mass index and inflammation: Pathways to depression and mental health problems in adolescents. Brain Behav. Immun..

[17] Lai J.S., Oldmeadow C., Hure A.J., McEvoy M., Hiles S.A., Boyle M., Attia J. (2016). Inflammation mediates the association between fatty acid intake and depression in older men and women. Nutr. Res..

[18] Bouchard L.C., Antoni M.H., Blomberg B.B., Stagl J.M., Gudenkauf L.M., Jutagir D.R., Diaz A., Lechner S., Glück S., Derhagopian R.P. (2016). Postsurgical Depressive Symptoms and Proinflammatory Cytokine Elevations in Women Undergoing Primary Treatment for Breast Cancer. Psychosom. Med..

[19] Wu T., Hsu F.C., Pierce J.P. (2020). Acid-Producing Diet and Depressive Symptoms among Breast Cancer Survivors: A Longitudinal Study. Cancers.

[20] Shivappa N., Steck S.E., Hurley T.G., Hussey J.R., Hébert J.R. (2014). Designing and developing a literature-derived, population-based dietary inflammatory index. Public Health Nutr..

[21] Shakya P.R., Melaku Y.A., Shivappa N., Hébert J.R., Adams R.J., Page A.J., Gill T.K. (2021). Dietary inflammatory index (DII^®^) and the risk of depression symptoms in adults. Clin. Nutr..

[22] Kheirouri S., Alizadeh M. (2019). Dietary Inflammatory Potential and the Risk of Incident Depression in Adults: A Systematic Review. Adv. Nutr..

[23] Li L., Yang Y., He J., Yi J., Wang Y., Zhang J., Zhu X. (2015). Emotional suppression and depressive symptoms in women newly diagnosed with early breast cancer. BMC Womens Health.

[24] Craig C.L., Marshall A.L., Sjöström M., Bauman A.E., Booth M.L., Ainsworth B.E., Pratt M., Ekelund U., Yngve A., Sallis J.F. (2003). International physical activity questionnaire: 12-country reliability and validity. Med. Sci. Sports Exerc..

[25] Radloff L.S. (1977). The CES-D Scale: A Self-Report Depression Scale for Research in the General Population. Appl. Psych. Meas..

[26] Hann D., Winter K., Jacobsen P. (1999). Measurement of depressive symptoms in cancer patients: Evaluation of the Center for Epidemiological Studies Depression Scale (CES-D). J. Psychosom. Res..

[27] Zung W.W. (1971). A rating instrument for anxiety disorders. Psychosomatics.

[28] Hébert J.R., Shivappa N., Wirth M.D., Hussey J.R., Hurley T.G. (2019). Perspective: The Dietary Inflammatory Index (DII)-Lessons Learned, Improvements Made, and Future Directions. Adv. Nutr..

[29] Imai C., Takimoto H., Fudono A., Tarui I., Aoyama T., Yago S., Okamitsu M., Sasaki S., Mizutani S., Miyasaka N. (2021). Application of the Nutrient-Rich Food Index 9.3 and the Dietary Inflammatory Index for Assessing Maternal Dietary Quality in Japan: A Single-Center Birth Cohort Study. Nutrients.

[30] Tingley D., Yamamoto T., Hirose K., Keele L., Imai K. (2014). mediation: R Package for Causal Mediation Analysis. J. Stat. Softw..

[31] Sarris J., Logan A.C., Akbaraly T.N., Amminger G.P., Balanzá-Martínez V., Freeman M.P., Hibbeln J., Matsuoka Y., Mischoulon D., Mizoue T. (2015). Nutritional medicine as mainstream in psychiatry. Lancet Psychiatry.

[32] Popa T.A., Ladea M. (2012). Nutrition and depression at the forefront of progress. J. Med. Life.

[33] Aly J., Engmann O. (2020). The Way to a Human’s Brain Goes through Their Stomach: Dietary Factors in Major Depressive Disorder. Front. Neurosci..

[34] Elstgeest L.E.M., Visser M., Penninx B., Colpo M., Bandinelli S., Brouwer I.A. (2019). Bidirectional associations between food groups and depressive symptoms: Longitudinal findings from the Invecchiare in Chianti (InCHIANTI) study. Br. J. Nutr..

[35] Kiecolt-Glaser J.K., Derry H.M., Fagundes C.P. (2015). Inflammation: Depression fans the flames and feasts on the heat. Am. J. Psychiatry.

[36] Perez-Cornago A., de la Iglesia R., Lopez-Legarrea P., Abete I., Navas-Carretero S., Lacunza C.I., Lahortiga F., Martinez-Gonzalez M.A., Martinez J.A., Zulet M.A. (2014). A decline in inflammation is associated with less depressive symptoms after a dietary intervention in metabolic syndrome patients: A longitudinal study. Nutr. J..

[37] Firth J., Veronese N., Cotter J., Shivappa N., Hebert J.R., Ee C., Smith L., Stubbs B., Jackson S.E., Sarris J. (2019). What Is the Role of Dietary Inflammation in Severe Mental Illness? A Review of Observational and Experimental Findings. Front. Psychiatry.

[38] Lucas M., Chocano-Bedoya P., Schulze M.B., Shulze M.B., Mirzaei F., O’Reilly É.J., Okereke O.I., Hu F.B., Willett W.C., Ascherio A. (2014). Inflammatory dietary pattern and risk of depression among women. Brain Behav. Immun..

[39] Manigault A.W., Kuhlman K.R., Irwin M.R., Cole S.W., Ganz P.A., Crespi C.M., Bower J.E. (2021). Vulnerability to inflammation-related depressive symptoms: Moderation by stress in women with breast cancer. Brain Behav. Immun..

[40] Miller A.H., Maletic V., Raison C.L. (2009). Inflammation and Its Discontents: The Role of Cytokines in the Pathophysiology of Major Depression. Biol. Psychiat..

[41] Raad T., Griffin A., George E.S., Larkin L., Fraser A., Kennedy N., Tierney A.C. (2021). Dietary Interventions with or without Omega-3 Supplementation for the Management of Rheumatoid Arthritis: A Systematic Review. Nutrients.

[42] Torres M.A., Pace T.W., Liu T., Felger J.C., Mister D., Doho G.H., Kohn J.N., Barsevick A.M., Long Q., Miller A.H. (2013). Predictors of depression in breast cancer patients treated with radiation: Role of prior chemotherapy and nuclear factor kappa B. Cancer.

[43] McFarland D.C., Doherty M., Atkinson T.M., O’Hanlon R., Breitbart W., Nelson C.J., Miller A.H. (2022). Cancer-related inflammation and depressive symptoms: Systematic review and meta-analysis. Cancer.

[44] Hart M.J., Torres S.J., McNaughton S.A., Milte C.M. (2021). Dietary patterns and associations with biomarkers of inflammation in adults: A systematic review of observational studies. Nutr. J..

[45] Huang S.C., Wei J.C., Wu D.J., Huang Y.C. (2010). Vitamin B(6) supplementation improves pro-inflammatory responses in patients with rheumatoid arthritis. Eur. J. Clin. Nutr..

[46] Mozaffari H., Askari M., Bellissimo N., Azadbakht L. (2021). Associations between dietary intake of B vitamins and cardiovascular risk factors in elderly men: A cross-sectional study. Int. J. Clin. Pract..

[47] Azarmanesh D., Bertone-Johnson E.R., Pearlman J., Liu Z., Carbone E.T. (2022). The dietary inflammatory index is inversely associated with depression, which is minimally mediated by C-reactive protein. Nutr. Res..

[48] Boomsma D.I., Willemsen G., Sullivan P.F., Heutink P., Meijer P., Sondervan D., Kluft C., Smit G., Nolen W.A., Zitman F.G. (2008). Genome-wide association of major depression: Description of samples for the GAIN Major Depressive Disorder Study: NTR and NESDA biobank projects. Eur. J. Hum. Genet..

[49] Kaster M.P., Gadotti V.M., Calixto J.B., Santos A.R.S., Rodrigues A.L.S. (2012). Depressive-like behavior induced by tumor necrosis factor-α in mice. Neuropharmacology.

[50] Tyring S., Gottlieb A., Papp K., Gordon K., Leonardi C., Wang A., Lalla D., Woolley M., Jahreis A., Zitnik R. (2006). Etanercept and clinical outcomes, fatigue, and depression in psoriasis: Double-blind placebo-controlled randomised phase III trial. Lancet.

[51] Guloksuz S., Wichers M., Kenis G., Russel M.G., Wauters A., Verkerk R., Arts B., van Os J. (2013). Depressive symptoms in Crohn’s disease: Relationship with immune activation and tryptophan availability. PLoS ONE.

[52] Han Q.Q., Yu J. (2014). Inflammation: A mechanism of depression?. Neurosci. Bull..

[53] Guebre-Egziabher F., Rabasa-Lhoret R., Bonnet F., Bastard J.P., Desage M., Skilton M.R., Vidal H., Laville M. (2008). Nutritional intervention to reduce the n-6/n-3 fatty acid ratio increases adiponectin concentration and fatty acid oxidation in healthy subjects. Eur. J. Clin. Nutr..

[54] Kenđel Jovanović G., Mrakovcic-Sutic I., Pavičić Žeželj S., Šuša B., Rahelić D., Klobučar Majanović S. (2020). The efficacy of an energy-restricted anti-inflammatory diet for the management of obesity in younger adults. Nutrients.

[55] Chmurzynska A., Muzsik A., Krzyżanowska-Jankowska P., Walkowiak J., Bajerska J. (2019). The effect of habitual fat intake, IL6 polymorphism, and different diet strategies on inflammation in postmenopausal women with central obesity. Nutrients.

[56] Sánchez-Villegas A., Martínez-González M.A., Estruch R., Salas-Salvadó J., Corella D., Covas M.I., Arós F., Romaguera D., Gómez-Gracia E., Lapetra J. (2013). Mediterranean dietary pattern and depression: The PREDIMED randomized trial. BMC Med..

[57] Parletta N., Zarnowiecki D., Cho J., Wilson A., Bogomolova S., Villani A., Itsiopoulos C., Niyonsenga T., Blunden S., Meyer B. (2019). A Mediterranean-style dietary intervention supplemented with fish oil improves diet quality and mental health in people with depression: A randomized controlled trial (HELFIMED). Nutr. Neurosci..

[58] Rapaport M.H., Nierenberg A.A., Schettler P.J., Kinkead B., Cardoos A., Walker R., Mischoulon D. (2016). Inflammation as a predictive biomarker for response to omega-3 fatty acids in major depressive disorder: A proof-of-concept study. Mol. Psychiatry.

[59] Su K.P., Lai H.C., Yang H.T., Su W.P., Peng C.Y., Chang J.P., Chang H.C., Pariante C.M. (2014). Omega-3 fatty acids in the prevention of interferon-alpha-induced depression: Results from a randomized, controlled trial. Biol. Psychiatry.

[60] Shelton R.C., Pencina M.J., Barrentine L.W., Ruiz J.A., Fava M., Zajecka J.M., Papakostas G.I. (2015). Association of obesity and inflammatory marker levels on treatment outcome: Results from a double-blind, randomized study of adjunctive L-methylfolate calcium in patients with MDD who are inadequate responders to SSRIs. J. Clin. Psychiatry.

[61] Corley J., Shivappa N., Hébert J.R., Starr J.M., Deary I.J. (2019). Associations between Dietary Inflammatory Index Scores and Inflammatory Biomarkers among Older Adults in the Lothian Birth Cohort 1936 Study. J. Nutr. Health Aging.

