# Supplementary material for: TNF-α Mediates the Association between Dietary Inflammatory Index and Depressive Symptoms in Breast Cancer
Source: Nutrients. 2022 Dec 24;15(1):84. doi: 10.3390/nu15010084 (PMC9823771; doi:10.3390/nu15010084)
Supplement: Supplementary file 1 [file nutrients-15-00084-s001.zip › nutrients-1981441-supplementary.pdf]

Table S1. Food parameters of energy-adjusted dietary inflammatory index (E-DII) used in this study.

Food parameter	Overall inflammatory effect score [a, b]	Global daily mean intake [a]	Global SD [a]	Global daily mean intake per 1000kcal energy intake [b]	Global SD per 1000kcal energy intake [b]
Vitamin B_{12} (μg)	0.106	5.15	2.70	2.50	1.31
Vitamin B_6 (mg)	−0.365	1.47	0.74	0.71	0.36
β-Carotene (μg)	−0.584	3718	1720	1808.37	836.58
Carbohydrate (g)	0.097	272.2	40.0	132.39	19.46
Cholesterol (mg)	0.110	279.4	51.2	135.89	24.9
Energy (kcal)	0.180	2056	338	1000	164.4
Total fat (g)	0.298	71.4	19.4	34.73	9.44
Dietary fiber (g)	−0.663	18.8	4.9	9.14	2.38
Folic acid (μg)	−0.190	273.0	70.7	132.78	34.39
Iron (mg)	0.032	13.35	3.71	6.49	1.80
Magnesium (mg)	−0.484	310.1	139.4	150.83	67.80
MUFAs (g)	−0.009	27.0	6.1	13.13	2.97
Niacin (mg)	−0.246	25.90	11.77	12.60	5.72
n-3 Fatty acids (g)	−0.436	1.06	1.06	0.52	0.52
n-6 Fatty acids (g)	−0.159	10.80	7.50	5.25	3.65
Protein (g)	0.021	79.4	13.9	38.62	6.76
PUFAs (g)	−0.337	13.88	3.76	6.75	1.83
Vitamin B_2 (mg)	−0.068	1.70	0.79	0.83	0.38
Saturated fatty acids (g)	0.373	28.6	8.0	13.91	3.89
Selenium (ug)	−0.191	67.0	25.1	32.59	12.21
Vitamin B_1 (mg)	−0.098	1.70	0.66	0.83	0.32
Vitamin A (ugRAE)	−0.401	983.9	518.6	478.55	252.24
Vitamin C (mg)	−0.424	118.2	43.46	57.49	21.14
Vitamin D (ug)	−0.446	6.26	2.21	3.04	1.07
Vitamin E (mg)	−0.419	8.73	1.49	4.25	0.72
Zinc (mg)	−0.313	9.84	2.19	4.79	1.07

MUFAs, monounsaturated fatty acids; PUFAs, polyunsaturated fatty acids; SD, standard deviation. [a] Shivappa N, Steck SE, Hurley TG, Hussey JR, Hébert JR. Designing and developing a literature-derived, population-based dietary inflammatory index. Public Health Nutr. 2014;17(8):1689-1696. [b] Imai C, Takimoto H, Fudono A, Tarui I, Aoyama T, Yago S, Okamitsu M, Sasaki S, Mizutani S, Miyasaka N, Sato N. Application of the Nutrient-Rich Food Index 9.3 and the Dietary Inflammatory Index for Assessing Maternal Dietary Quality in Japan: A Single-Center Birth Cohort Study. Nutrients. 2021;13(8):2854.

Table S2. Univariate logistic regression analysis of factors influencing depressive symptoms in breast cancer patients (n = 220).

**Variables**	**Univariate**		
	**OR**	**95% CI**	***p*-value**
E-DII score	1.50	1.24, 1.83	<0.001
SAS score	1.33	1.23, 1.44	<0.001
Education level			
Primary school and below	1.00	Reference	
Middle school	0.39	0.17, 0.86	0.020
High school/ secondary school	0.42	0.16, 1.11	0.080
Junior college or above	0.28	0.11, 0.72	0.008
Residence			
Rural areas	1.00	Reference	
Towns	0.34	0.15, 0.80	0.013
Urban areas	0.64	0.33, 1.27	0.202
Family monthly income (RMB)			
<2000	1.00	Reference	
2000-5000	0.55	0.23, 1.34	0.189
>5000	0.30	0.12, 0.78	0.013
Physical activity level			
Low	1.00	Reference	
Moderate	0.42	0.23, 0.79	0.007
High	0.19	0.02, 1.58	0.124
Drinking status			
Former/Current	1.00	Reference	
Never	0.14	0.03, 0.74	0.021

E-DII, energy-adjusted Dietary Inflammatory Index; SAS, Self-rating Anxiety Scale.

Table S3. Multivariate logistic regression analysis of factors influencing depressive symptoms in breast cancer patients (n = 220).

Variables	Multivariate		
	OR	95% CI	*p*-value
E-DII score	1.52	1.16, 1.99	0.003
SAS score	1.36	1.23, 1.50	<0.001
Education level			
Primary school and below	1.00	Reference	
Middle school	0.90	0.28, 2.97	0.863
High school/ secondary school	0.68	0.15, 3.17	0.627
Junior college or above	0.43	0.10, 1.90	0.263
Residence			
Rural areas	1.00	Reference	
Towns	0.60	0.17, 2.15	0.434
Urban areas	0.70	0.23, 2.17	0.540
Family monthly income (RMB)			
<2000	1.00	Reference	
2000-5000	0.78	0.20, 3.03	0.725
>5000	0.86	0.20, 3.76	0.842
Physical activity level			
Low	1.00	Reference	
Moderate	0.76	0.31, 1.85	0.547
High	0.65	0.05, 7.74	0.731
Drinking status			
Former/Current	1.00	Reference	
Never	0.09	0.01, 0.71	0.022

E-DII, energy-adjusted Dietary Inflammatory Index; SAS, Self-rating Anxiety Scale.

Table S4. Dietary nutrient intakes of breast cancer patients in different E-DII tertiles.

Variables	Lowest tertile (n = 74)	Middle tertile (n = 73)	Highest tertile (n = 73)	p-value
Energy (kcal/d)	1510.0 (1267.3, 1752.8)	1609.0 (1261.5, 1919.5)	1587.5 (1287.0, 1844.5)	0.437
Protein (g/d)	81.9 (55.7, 107.1)	70.4 (54.3, 91.9)	64.2 (50.7, 84.1)	0.081
Total fat (g/d)	62.0 (50.8, 74.9)	58.8 (47.7, 78.3)	68.7 (54.4, 80.2)	0.155
Carbohydrate (g/d)	157.6 (129.1, 202.0)	188.1 (149.0, 249.4)	178.7 (136.3, 222.1)	0.017
Dietary fiber (g/d)	13.2 (10.3, 20.1)	13.9 (9.1, 21.1)	9.4 (7.4, 12.9)	<0.001
Cholesterol (mg/d)	605.0 (423.0, 1028.3)	566.0 (356.5, 789.5)	535.0 (245.5, 737.0)	0.063
Vitamin A (μgRAE/d)	637.0 (471.5, 930.5)	500 (358.5, 624.0)	363.0 (258.0, 543.5)	<0.001
Vitamin B_1 (mg/d)	0.8 (0.6, 1.0)	0.8 (0.6, 1.0)	0.8 (0.6, 1.0)	0.488
Vitamin B_2 (mg/d)	1.3 (0.8, 1.7)	1.0 (0.8, 1.5)	0.9 (0.7, 1.3)	0.002
Vitamin B_6 (mg/d)	0.3 (0.2, 0.4)	0.2 (0.1, 0.3)	0.1 (0.1, 0.2)	<0.001
Vitamin B_{12} (μg/d)	0.2 (0.1, 0.2)	0.2 (0.1, 0.2)	0.2 (0.1, 0.2)	0.360
Vitamin C (mg/d)	221.9 (144.4, 307.3)	180.0 (132.6, 227.2)	91.5 (61.5, 136.1)	<0.001
Vitamin D (μg/d)	2.8 (0.4, 8.4)	1.0 (0.0, 4.5)	0.5 (0.0, 2.1)	<0.001
Vitamin E (mg/d)	30.1 (24.3, 35.7)	26.2 (20.6, 33.6)	21.1 (13.8, 26.0)	<0.001
Folic acid (μg/d)	172.5 (121.7, 243.0)	144.8 (94.9, 193.9)	91.2 (62.7, 133.3)	<0.001
Niacin (mg/d)	16.4 (11.7, 21.9)	14.1 (12.0, 19.5)	12.0 (9.4, 17.9)	0.004
β-Carotene (mg/d)	2.7 (1.7, 3.9)	1.6 (0.9, 2.7)	0.9 (0.4, 1.5)	<0.001
Magnesium (mg/d)	347.0 (285.5, 454.0)	320.0 (233.5, 395.0)	235.0 (193.0, 325.5)	<0.001
Iron (mg/d)	20.2 (16.2, 25.0)	18.1 (13.9, 22.3)	16.5 (12.6, 22.6)	0.007
Zinc (mg/d)	11.1 (8.1, 14.5)	10.1 (7.8, 12.8)	8.5 (6.3, 10.6)	<0.001
Selenium (μg/d)	72.9 (38.4, 101.2)	56.5 (33.0, 85.9)	40.3 (29.4, 60.2)	<0.001
SFA (g/d)	12.9 (9.7, 18.9)	14.3 (10.5, 17.4)	17.2 (12.5, 20.8)	0.002
MUFA (g/d)	22.8 (17.6, 28.7)	23.1 (15.6, 30.5)	25.0 (20.4, 32.5)	0.082
PUFA (g/d)	15.9 (12.8, 19.5)	14.1 (9.7, 17.9)	12.7 (9.3, 17.9)	0.005
Omega-3 fatty acids (g/d)	0.2 (0.2, 0.3)	0.2 (0.1, 0.2)	0.2 (0.1, 0.2)	0.003
Omega-6 fatty acids (g/d)	1.4 (1.0, 1.7)	1.2 (0.7, 1.6)	1.1 (0.7, 1.5)	0.005

Data are shown as median (25th, 75th percentile). E-DII, energy-adjusted Dietary Inflammatory Index; SFA, saturated fatty acids; MUFA, monounsaturated fatty acids; PUFA, polyunsaturated fatty acids. E-DII tertile ranges: the lowest tertile (−4.72 ~ −0.92), the middle tertile (−0.91 ~ 0.63), and the highest tertile (0.64 ~ 3.72).

Table S5. Association between E-DII and its components with plasma inflammatory markers (n = 123).

Variables	CRP	TNF-α	IL-6	IL-1β
E-DII	0.29 (0.01, 0.05) *	0.22 (0.04, 0.41) *	0.14 (−0.02, 0.19)	0.17 (−0.004, 0.29)
Carbohydrate	−0.03 (−0.001, 0.001)	0.03 (−0.01, 0.01)	0.02 (−0.003, 0.004)	0.01 (−0.004, 0.005)
Vitamin A	−0.13 (−0.02, 0.003)	−0.02 (−0.001, 0.001)	0.001 (−0.001, 0.001)	−0.08 (−0.11, 0.04)
Vitamin B_2	0.01 (−0.06, 0.06)	−0.05 (−0.69, 0.38)	−0.04 (−0.37, 0.23)	−0.16 (−0.80, 0.04)
Vitamin B_6	−0.22 (−0.46, −0.06) *	−0.17 (−3.51, 0.10)	−0.01 (−1.10, 0.98)	−0.13 (−2.54, 0.38)
Folic acid	−0.06 (−0.001, 0.001)	−0.01 (−0.01, 0.004)	0.03 (−0.002, 0.003)	0.01 (−0.003, 0.004)
β-Carotene	−0.17 (−0.06, 0.001)	−0.16 (−0.48, 0.03)	−0.11 (−0.24, 0.06)	−0.13 (−0.36, 0.05)
Iron	−0.04 (−0.01, 0.004)	−0.03 (−0.05, 0.04)	0.10 (−0.01, 0.04)	−0.07 (−0.05, 0.02)
Zinc	−0.05 (−0.12, 0.01)	−0.09 (−0.12, 0.04)	0.10 (−0.02, 0.07)	−0.15 (−0.12, 0.01)

Data are shown as beta coefficient and 95% confidence interval. Multiple linear regression analysis was used after adjusting for age, SAS scores and drinking status. * $p < 0.05$.
E-DII, energy-adjusted Dietary Inflammatory Index; CRP, C-reactive protein; TNF-α, tumor necrosis factor α; IL-6, interleukin 6; IL-1β, interleukin 1β.
